# Supplementary material for: Radiomics from Mesorectal Blood Vessels and Lymph Nodes: A Novel Prognostic Predictor for Rectal Cancer with Neoadjuvant Therapy
Source: Diagnostics (Basel). 2023 Jun 6;13(12):1987. doi: 10.3390/diagnostics13121987 (PMC10297215; doi:10.3390/diagnostics13121987)
Supplement: Supplementary file 1 [file diagnostics-13-01987-s001.zip › diagnostics-2376587-supplementary.pdf]

diagnostics

MDPI

Supplementary Materials

Table S1 Parameters of MR scanners used in this study.

	MR 750	MR Prisma
Reptetion time (ms)	4433	5740
Echo time (ms)	115	101
Section thickness (mm)	4	3
Section space (mm)	0.8	0
Field of view (mm2)	200×200	200×200
Acquisition Time (s)	217	155

Table S2. Inter–reader ICCs of radiomics features for each ROI.

ROI	ROI_{ITU}	ROI_{PTU_2mm}	ROI_{PTU_4mm}	ROI_{PTU_6mm}	ROI_{MR_F}	ROI_{MR_BVLN}
ICC>0.80	1787	930	1186	1553	1417	1380
ICC_{mean}	0.83	0.67	0.71	0.76	0.76	0.76
ICC_{median}	0.90	0.71	0.75	0.82	0.82	0.82
ICC_{1stQu}	0.81	0.57	0.63	0.73	0.68	0.68
ICC_{3rdQu}	0.95	0.83	0.86	0.90	0.90	0.90

ICC, intraclass correlation coefficient; ICC_{1stQu}, the first quartile of ICC; ICC_{3rdQu}, the third quartile of ICC.

Table S3. Total number of features and remaining features after feature selection for each roi or roi combination.

ROI	Total Number	ICC	Variance Threshold	Univariate Selection	LASSO
ROI_{ITU}	2264	1787	1344	422	16
ROI_{PTU_2mm}	2264	930	708	356	20
ROI_{PTU_4mm}	2264	1186	885	489	23
ROI_{PTU_6mm}	2264	1553	1200	697	16
ROI_{MR_F}	2264	1417	1045	13	10
ROI_{MR_BVLN}	2264	1380	1058	384	11
ROI_{ITU}+ROI_{PTU_2mm}	4528	2717	2052	778	22
ROI_{ITU}+ROI_{PTU_4mm}	4528	2973	2229	911	24
ROI_{ITU}+ROI_{PTU_6mm}	4528	3340	2544	1119	21
ROI_{ITU}+ROI_{MR_F}	4528	3204	2389	435	25
ROI_{ITU}+ROI_{MR_BVLN}	4528	3167	2402	806	28
ROI_{ITU}+ROI_{MR_F} +ROI_{MR_BVLN}	6792	4584	3447	819	24

ICC, intraclass correlation coefficient; LASSO, least absolute shrinkage and selection operator

Table S4. The remaining features of ROI$_{ITU}$ after feature selection

Filter	Feature Category	Feature Name
Wavelet	GLRLM	wavelet hll ShortRunLowGrayLevelEmphasis
LaplacianSharpening	GLDM	SmallDependenceHighGrayLevelEmphasis
DiscreteGaussian	GLRLM	ShortRunLowGrayLevelEmphasis
Log	GLDM	log sigma 1.0 mm 3d DependenceEntropy
SpeckleNoise	GLDM	SmallDependenceEmphasis
DiscreteGaussian	GLDM	SmallDependenceLowGrayLevelEmphasis
DiscreteGaussian	GLSZM	graylevelnonuniformity
Log	GLDM	log sigma 4.0 mm 3d LargeDependenceHighgrayLevelEmphasis
BinomialBlurimage	GLSZM	Large Area Emphasis
Normalize	GLCM	ClusterShade
Log	GLRLM	log sigma 1.0 mm 3d Runvariance
Log	GLSZM	log sigma 4.0 mm 3d GrayLevelNonuniformity
Log	GLRLM	log sigma 4.0 mm 3d LongRunHighGrayLevelEmphasis
Original	GLSZM	SizeZoneNonuniformity
ShotNoise	GLRLM	ShortRunHighHrayLevelEmphasis
BinomialBlurimage	Firstorder	Range

Table S5. The remaining features of ROI$_{PTU_2mm}$ after feature selection

Filter	Feature Category	Feature Name
Wavelet	GLCM	wavelet lhl MaximumProbability
Log	Firstorder	log sigma 1.0.mm 3d Kurtosis
Shotnoise	NGTDM	Strength
Original	Shape	MajorAxislength
Recursivegaussian	GLCM	Imc1
Mean	GLCM	Imc1
Mean	NGTDM	Coarseness
Original	NGTDM	Strength
Shotnoise	NGTDM	Coarseness
Original	NGTDM	Coarseness
Curvatureflow	Firstorder	90percentile
Specklenoise	Firstorder	90percentile
Recursivegaussian	GLSZM	LargeAreaHighGrayLevelEmphasis
Log	GLRLM	log sigma 1.0 mm 3d Runvariance
Mean	GLCM	Correlation
Wavelet	GLRLM	wavelet hlh RunVariance
Log	Firstorder	log sigma 1.0.mm 3d Interquartilerange
Log	GLSZM	log sigma 0.5 mm 3d Graylevelnonuniformity
Wavelet	GLSZM	wavelet lhl LargeAreaLowGrayLevelEmphasis
Discretegaussian	GLSZM	lowgraylevelzoneemphasis

Table S6. The remaining features of ROI$_{PTU_4mm}$ after feature selection

Filter	Feature Category	Feature Name
Original	Shape	Maximum3dDiameter
Boxmean	Firstorder	Kurtosis
Original	Shape	Sphericity
Laplaciansharpening	GLDM	LargeDependenceLowgrayLevelEmphasis
Curvatureflow	NGTDM	Coarseness
Wavelet	Firstorder	wavelet hll Median
Normalize	NGTDM	Strength
Wavelet	Firstorder	wavelet hll 10percentile
Normalize	GLCM	Imc1
Original	GLSZM	GrayLevelNonuniformity
Log	Firstorder	log sigma 1.0 mm 3d Kurtosis
Log	Firstorder	log sigma 4.0 mm 3d 90percentile
Curvatureflow	GLSZM	LowGrayLevelZoneEmphasis
Boxsigmaimage	GLRLM	RunLengthNonuniformity
Wavelet	GLSZM	wavelet lll LargeAreaHighGrayLevelEmphasis
Log	GLDM	log sigma 4.0 mm 3d LargeDependenceLowGrayLevelEmphasis
Additivegaussian-noise	GLSZM	GrayLevelNonuniformity
Log	GLRLM	log sigma 1.0 mm 3d RunVariance
Curvatureflow	GLSZM	LargeAreaHighGrayLevelEmphasis
Log	GLSZM	log sigma 0.5 mm 3d GrayLevelNonuniformity
Wavelet	GLSZM	wavelet hll GrayLevelNonuniformity
Discretegaussian	GLCM	IDMN
Recursivegaussian	GLCM	Correlation

Table S7. The remaining features of ROI$_{PTU_6mm}$ after feature selection

Filter	Feature Category	Feature Name
Recursivegaussian	GLCM	Imc1
Normalize	GLSZM	HighGrayLevelZoneEmphasis
Laplaciansharpening	GLSZM	HighGrayLevelZoneEmphasis
Original	Shape	Sphericity
Log	NGTDM	log sigma 2.0 mm 3d Coarseness
Log	GLRLM	log sigma 4.0 mm 3d ShortRunLowGrayLEvelEmphasis
Wavelet	GLRLM	wavelet hll RunVariance
Wavelet	GLCM	wavelet hll ClusterShade
Curvatureflow	GLSZM	ZoneVariance
Log	GLSZM	log sigma 0.5 mm 3d GrayLevelNonuniformity
Wavelet	GLSZM	wavelet hll GrayLevelNonuniformity
Wavelet	GLSZM	wavelet lll SizeZoneNonuniformity
Log	GLRLM	log sigma 4.0 mm 3d ShortRunHighGrayLevelEmphasis
Wavelet	GLCM	wavelet hll IDMN
Log	GLSZM	log sigma 1.0 mm 3d GrayLevelNonuniformity
Curvatureflow	Firstorder	Minimum

Table S8. The remaining features of ROI_{MR_F} after feature selection

Filter	Feature Category	Feature Name
Curvatureflow	GLRLM	GrayLevelNonuniformity
Original	GLRLM	GrayLevelNonuniformity
Log	GLRLM	log sigma 4.0 mm 3d GrayLevelNonuniformity
Wavelet	Firstorder	wavelet hhl Skewness
Recursivegaussian	GLCM	Imc1
Additivegaussian-noise	GLSZM	GrayLevelNonuniformity
Curvatureflow	GLRLM	RunLengthNonuniformity
Binomialblurimage	GLRLM	GrayLevelNonuniformity
Mean	GLRLM	GrayLevelNonuniformity
Recursivegaussian	GLRLM	GRayLevelNonuniformity

Table S9. The remaining features of ROI_{MR_BVLN} after feature selection

Filter	Feature Category	Feature Name
Wavelet	Firstorder	wavelet lhl 90percentile
Laplaciansharpening	GLSZM	HighGrayLevelZoneEmphasis
Log	Firstorder	log sigma 1.0 mm 3d Median
Log	Firstorder	log sigma 2.0 mm 3d RootMeanSquared
Specklenoise	Firstorder	10percentile
Original	GLSZM	LowGrayLevelZoneEmphasis
Log	GLCM	log sigma 1.0 mm 3d Imc2
Wavelet	GLCM	wavelet llh ClusterProminence
Additivegaussian-noise	GLSZM	GrayLevelNonuniformity
Boxsigmaimage	NGTDM	Busyness
Log	Firstorder	log sigma 2.0 mm 3d Range

Table S10. The remaining features of ROI_{ITU}+ROI_{PTU_2mm} after feature selection

ROI	Filter	Feature Category	Feature Name
ROI_{ITU}	Binomialblurimage	Firstorder	Range
	Discretegaussian	GLDM	DependenceVariance
	Discretegaussian	GLRLM	ShortRunLowGrayLevelEmphasis
	Laplaciansharpen-ing	GLDM	SmallDependenceHighGrayLevelEmphasis
	Log	GLDM	log sigma 1.0 mm 3d Dependenceentropy
	Log	GLRLM	log sigma 1.0 mm 3d RunVariance
	Mean	GLSZM	ZonePercentage
	Original	GLSZM	SizeZoneNonuniformity
	Wavelet	GLRLM	wavelet hll ShortRunLowGrayLevelEmphasis
ROI_{PTU_2mm}	Boxmean	GLRLM	RunEntropy
	Discretegaussian	GLSZM	LowGrayLevelZoneEmphasis
	Log	Firstorder	log sigma 1.0 mm 3d InterquartileRange
	Log	Firstorder	log sigma 1.0 mm 3d Kurtosis

Log	GLDM	log sigma 2.0 mm 3d DependenceNonuniformityNormalized
Mean	GLCM	Correlation
Recursivegaussian	GLCM	Imc1
Shotnoise	NGTDM	Coarseness
Shotnoise	NGTDM	Strength
Specklenoise	Firstorder	90percentile
Wavelet	Firstorder	wavelet lll 90percentile
Wavelet	GLCM	wavelet lhl Maximumprobability
Wavelet	GLSZM	wavelet lhl LargeAreaLowGrayLevelEmphasis

Table S11. The remaining features of ROI_{ITU}+ROI_{PTU_4mm} after feature selection

ROI	Filter	Feature Category	Feature Name
ROI_{ITU}	Binomialblurimage	Firstorder	Range
	Binomialblurimage	GLSZM	LargeAreaEmphasis
	Discretegaussian	GLDM	DependenceVariance
	Discretegaussian	GLDM	SmallDependenceLowGrayLevelEmphasis
	Discretegaussian	GLRLM	ShortRunLowGrayLevelEmphasis
	Laplaciansharpening	GLDM	SmallDependenceHighGrayLevelEmphasis
	Log	GLDM	log sigma 1.0 mm 3d DependenceEntropy
	Log	GLRLM	log sigma 1.0 mm 3d RunVariance
	Mean	GLSZM	ZonePercentage
	Shotnoise	GLRLM	ShortRunHighGrayLevelEmphasis
	Wavelet	GLDM	wavelet lhl SmallDependenceEmphasis
	Wavelet	GLRLM	wavelet hll ShortRunLowGrayLevelEmphasis
ROI_{PTU_4mm}	Additivegaussian-noise	GLSZM	GrayLevelNonUniformity
	Boxmean	Firstorder	Kurtosis
	Boxsigmaimage	GLSZM	SizeZoneNonuniformity
	Curvatureflow	NGTDM	Coarseness
	Laplaciansharpening	GLDM	LargeDependenceLowGrayLevelEmphasis
	Log	GLDM	log sigma 4.0 mm 3d– LargeDependenceLowGrayLevelEmphasis
	Log	GLSZM	log sigma 0.5 mm 3d GrayLevelNonuniformity
	Normalize	NGTDM	Strength
	Original	Shape	Sphericity
	Recursivegaussian	GLCM	Correlation
	Wavelet	Firstorder	wavelet hll Median
	Wavelet	GLSZM	wavelet hll GrayLevelNonuniformity

Table S12. The remaining features of ROI_{ITU}+ROI_{PTU_6mm} after feature selection

ROI	Filter	Feature Category	Feature Name
ROI_{ITU}	Binomialblurimage	Firstorder	Range
	Discretegaussian	GLDM	DependenceVariance
	Discretegaussian	GLDM	SmallDependenceLowGrayLevelEmphasis
	Discretegaussian	GLRLM	ShortRunLowGrayLevelEmphasis
	Log	GLDM	log sigma 1.0 mm 3d DependenceEntropy
	Log	GLRLM	log sigma 1.0 mm 3d RunVariance
	Mean	GLSZM	ZonePercentage
	Shotnoise	GLRLM	ShortRunHighGrayLevelEmphasis
	Specklenoise	GLDM	SmallDependenceEmphasis
	Wavelet	GLRLM	wavelet hll ShortRunLowGrayLevelEmphasis
ROI_{PTU_6mm}	Curvatureflow	Firstorder	Minimum
	Laplaciansharpen-ing	GLSZM	HighGrayLevelZoneEmphasis
	Log	GLRLM	log sigma 4.0 mm 3d ShortRunHighGrayLevelEmphasis
	Log	GLSZM	log sigma 0.5 mm 3d GrayLevelNonuniformity
	Log	GLSZM	log sigma 1.0 mm 3d GrayLevelNonuniformity
	Normalize	GLSZM	HighGrayLevelZoneEmphasis
	Recursivegaussian	GLCM	Imc1
	Wavelet	GLCM	wavelet hll Clustershade
	Wavelet	GLCM	wavelet hll IDMN
	Wavelet	GLSZM	wavelet hll GrayLevelNonuniformity
	Wavelet	GLSZM	wavelet lll SIzeZoneNonuniformity

Table S13. The remaining features of ROI_{ITU}+ROI_{MR_F} after feature selection

ROI	Filter	Feature Category	Feature Name
ROI_{ITU}	Additivegaussian-noise	GLDM	SmallDependenceLowGrayLevelEmphasis
	Binomialblurimage	GLSZM	LargeAreaEmphasis
	Binomialblurimage	Firstorder	Range
	Boxsigmaimage	GLCM	Imc1
	Boxsigmaimage	Firstorder	RobustMeanAbsoluteDeviation
	Discretegaussian	GLRLM	ShortRunLowGrayLevelEmphasis
	Discretegaussian	GLDM	DependenceVariance
	Discretegaussian	GLSZM	GrayLevelNonuniformity
	Laplaciansharpening	GLDM	SmallDependenceHighGrayLevelEmphasis
	Log	GLDM	log sigma 1.0 mm 3d DependenceEntropy
	Log	GLSZM	log sigma 2.0 mm 3d HighGrayLevelZoneEmphasis
	Log	GLDM	log sigma 4.0 mm 3d LargeDependenceHighGrayLevelEm-phasis
	Log	GLSZM	log sigma 4 0 mm 3d GrayLevelNonuniformity
	Log	GLRLM	log sigma 1.0 mm 3d RunVariance
	Mean	GLSZM	ZonePercentage
	Mean	GLCM	Imc2
	Normalize	GLCM	ClusterShade
	Original	GLRLM	ShortRunLowGrayLevelEmphasis

	Original	GLSZM	SizeZoneNonuniformity
	Shotnoise	GLRLM	ShortRunHighGrayLevelEmphasis
	Wavelet	GLRLM	wavelet hll ShortRunLowGrayLevelEmphasis
	Wavelet	GLDM	wavelet llh LargeDependenceLowGrayLevelEmphasis
ROI_{MR_F}	Recursivegaussian	GLCM	Imc1
	Recursivegaussian	GLRLM	RunLengthNonuniformity
	Wavelet	Firstorder	wavelet hhl Skewness

Table S14. The remaining features of ROI_{ITU}+ROI_{MR_BVLN} after feature selection

ROI	Filter	Feature Category	Feature Name
ROI_{ITU}	Wavelet	GLDM	wavelet llh LargeDependenceLowGrayLevelEmphasis
	Wavelet	GLRLM	wavelet hll ShortRunLowGrayLevelEmphasis
	Specklenoise	GLDM	SmallDependenceEmphasis
	Shotnoise	GLRLM	ShortRunHighGrayLevelEmphasis
	Original	GLRLM	ShortRunLowGrayLevelEmphasis
	Original	GLSZM	SizeZoneNonuniformity
	Normalize	GLCM	ClusterShade
	Log	GLDM	log sigma 1.0 mm 3d DependenceEntropy
	Log	GLRLM	log sigma 1.0 mm 3d RunVariance
	Log	GLSZM	log sigma 4.0 mm 3d GrayLevelNonuniformity
	Log	GLDM	log sigma 4.0 mm 3d LargeDependenceHighGrayLevelEmphasis
	Laplaciansharpening	GLDM	SmallDependenceHighGrayLevelEmphasis
	Discretegaussian	GLDM	DependenceVariance
	Discretegaussian	GLRLM	ShortRunLowGrayLevelEmphasis
	Discretegaussian	GLSZM	GrayLevelNonuniformity
ROI_{MR_BVLN}	Wavelet	Firstorder	wavelet lhl 90percentile
	Wavelet	Firstorder	wavelet llh Entropy
	Wavelet	GLDM	wavelet hhl DependenceEntropy
	Wavelet	GLDM	wavelet hll LargeDependenceEmphasis
	Wavelet	GLSZM	wavelet lll SizeZoneNonuniformity
	Specklenoise	GLDM	SmallDependenceLowGrayLevelEmphasis
	Original	GLSZM	HighGrayLevelZoneEmphasis
	Log	Firstorder	log sigma 2.0 mm 3d Range
	Log	Firstorder	log sigma 2.0 mm 3d RootMeanSquared
	Log	GLCM	log sigma 0.5 mm 3d DifferenceEntropy
	Log	GLCM	log sigma 1.0 mm 3d Imc2
	Laplaciansharpening	GLSZM	HighGrayLevelZoneEmphasis
	Boxsigmaimage	NGTDM	Busyness

Table S15. The remaining features of ROI_{ITU}+ROI_{MR_F}+ROI_{MR_BVLN} after feature selection

ROI	Filter	Feature Category	Feature Name
ROI_{ITU}	Discretegaussian	GLDM	DependenceVariance
	Discretegaussian	GLRLM	ShortRunLowGrayLevelEmphasis
	Laplaciansharpening	GLDM	SmallDependenceHighGrayLevelEmphasis
	Log	GLDM	log sigma 4.0 mm 3d LargeDependenceHighGrayLevelEmphasis
	Log	GLRLM	log sigma 1.0 mm 3d RunVariance
	Log	GLSZM	log sigma 4.0 mm 3d GrayLevelNonuniformity
	Normalize	GLCM	ClusterShade
	Original	GLRLM	ShortRunLowGrayLevelEmphasis
	Specklenoise	GLDM	SmallDependenceEmphasis
	Wavelet	GLDM	wavelet llh LargeDependenceLowGrayLevelEmphasis
	Wavelet	GLRLM	wavelet hll ShortRunLowGrayLevelEmphasis
ROI_{MR_F}	Recursivegaussian	GLCM	Imc1
	Wavelet	Firstorder	wavelet hhl Skewness
ROI_{MR_BVLN}	Additivegaussian-noise	GLSZM	GrayLevelNonuniformity
	Boxsigmaimage	NGTDM	Busyness
	Laplaciansharpening	GLSZM	HighGrayLevelZoneEmphasis
	Log	Firstorder	log sigma 2.0 mm 3d Range
	Log	Firstorder	log sigma 2.0 mm 3d RootMeanSquared
	Log	GLCM	log sigma 1.0 mm 3d Imc2
	Original	GLSZM	HighGrayLevelZoneEmphasis
	Specklenoise	GLDM	SmallDependenceLowGrayLevelEmphasis
	Wavelet	Firstorder	wavelet lhl 90percentile
	Wavelet	Firstorder	wavelet llh Entropy
	Wavelet	GLSZM	wavelet lll SizeZoneNonuniformity

Table S16. The univariate and multivariate analysis of clinical factors in the training cohort.

Features	pPR (n=73[a])	pGR (n=94)	univariate analysis		multivariate analysis	
			OR (95%CI)	P	OR (95%CI)	P
DTAV						
≤4cm	29 (39.7%)	13 (13.8%)				
>4cm	44 (60.3%)	81 (86.2%)	4.11 (1.94–8.69)	<0.001	4.41 (2.04–9.52)	<0.001
MRF						
Negative	42 (57.5%)	67 (71.3%)				
Positive	31 (42.5%)	27 (28.7%)	0.55 (0.29–1.04)	0.066		
PLT	254.2±69.8	229.3±63.0	0.99 (0.99–1.00)	0.019		
PLR	164.2±82.9	135.6±50.4	0.99 (0.99–1.00)	0.011	0.99 (0.99–1.00)	0.008

[a] Mean ± standard deviation; number (percentage).

Abbreviations: pPR, poor pathological response; pGR, good pathological response; OR, odds ratio; CI, confidence interval; MRF, mesorectal fascia involvement; PLT, platelet count; PLR, platelet–to–lymphocyte ratio.

Table S17. The optimal cut–off values and their corresponding log–rank statistics for each variable to differentiate disease–free survival.

Continuous variables	cutpoint	log–rank statistics
Age (year)	48	1.632411
BMI (kg/m^2)	24.60973	1.562459
WBC (×10^9/L)	5.22	1.162395
HGB (g/L)	131	1.309876
PLT (×10^9/L)	263	1.444649
Lymphocyte (×10^9/L)	2.27	1.323995
Neutrophil (×10^9/L)	2.59	1.976648
Eosinophilic granulocyte (×10^9/L)	0.28	2.08096
Monocyte (×10^9/L)	0.54	0.65817
NLR	2.162162	1.546421
LMR	2.641026	2.210821
PLR	88.07947	1.70645
Radscore	0.68787	2.252407

BMI, body mass index; WBC, white blood cell count; HGB, hemoglobin level; PLT, platelet count; NLR, neutro–phil–to–lymphocyte ratio; LMR, lymphocyte–to–monocyte ratio; PLR, plate–let–to–lymphocyte ratio.

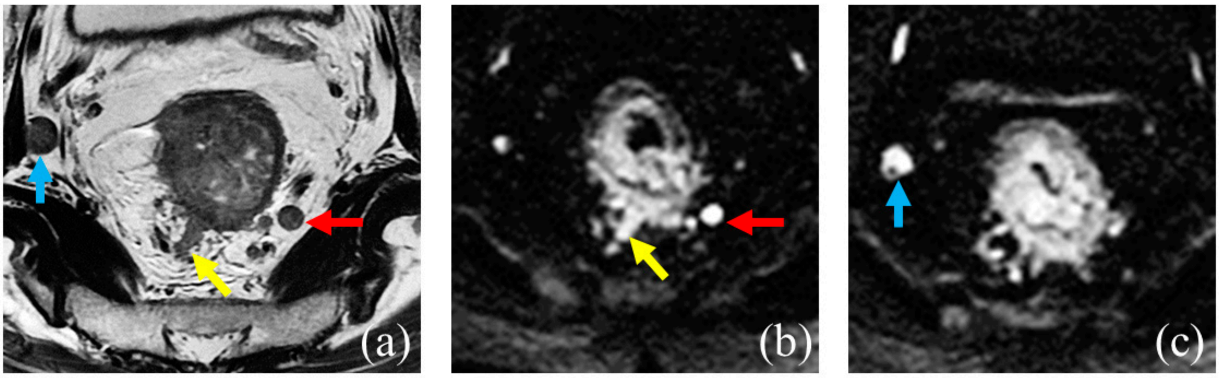

Figure S1. A 35–year–old male with locally advanced rectal cancer. His imaging studies include: (a) an oblique axial high–resolution T2–weighted image; (b)~(c) axial diffusion–weighted imaging (DWI) images. The red arrow points to a metastatic lymph node (DWI high signal) that is less than 1mm away from the mesorectal fascia, indicating mesorectal fascia involvement (MRF). The yellow arrow shows tumor signal in the perirectal vessels, indicating extramural vascular invasion (EMVI). The blue arrow marks enlarged lymph nodes outside the mesorectal fascia, with a short diameter >5mm and DWI high signal, indicating lateral pelvic lymph node metastasis (LPLN).

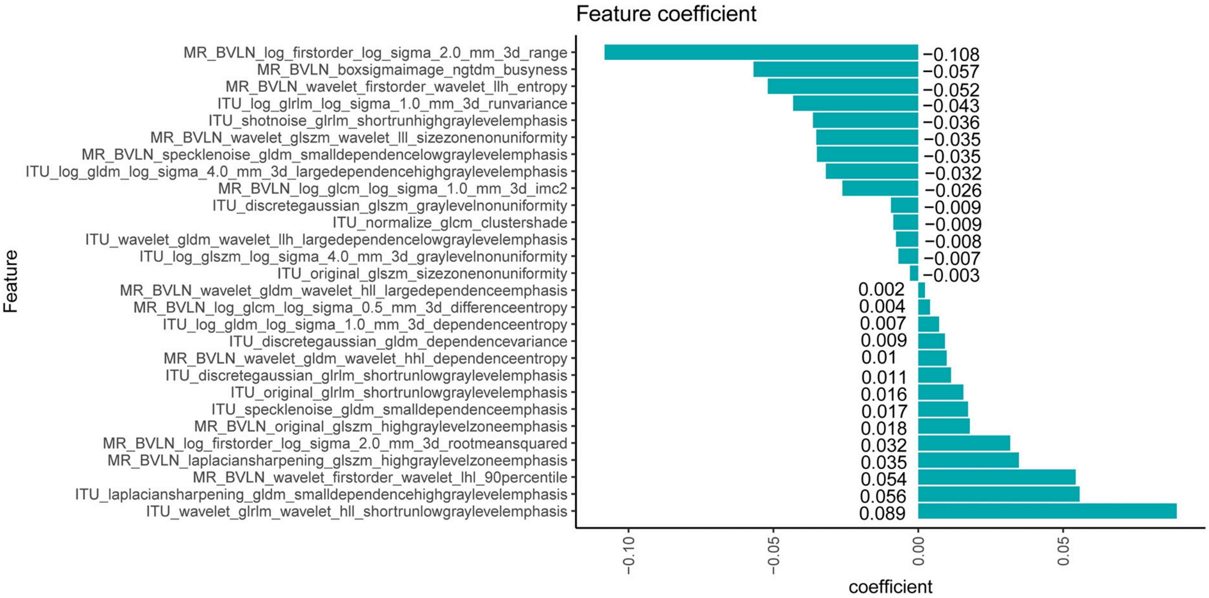

Figure S2. The LASSO regression result and feature coefficients for ROI_{ITU}+ROI_{MR_BVLN}. LASSO, least absolute shrinkage and selection operator.
